# Seabird and pinniped shape soil bacterial communities of their settlements in Cape Shirreff, Antarctica

**DOI:** 10.1371/journal.pone.0209887

**Published:** 2019-01-09

**Authors:** Lía Ramírez-Fernández, Nicole Trefault, Margarita Carú, Julieta Orlando

**Affiliations:** 1 Laboratory of Microbial Ecology, Department of Ecological Sciences, Faculty of Sciences, Universidad de Chile, Santiago, Chile; 2 Centre for Genomics, Ecology and Environment (GEMA), Faculty of Sciences, Universidad Mayor, Santiago, Chile; Uniwersytet Gdanski, POLAND

## Abstract

Seabirds and pinnipeds play an important role in biogeochemical cycling by transferring nutrients from aquatic to terrestrial environments. Indeed, soils rich in animal depositions have generally high organic carbon, nitrogen and phosphorus contents. Several studies have assessed bacterial diversity in Antarctic soils influenced by marine animals; however most have been conducted in areas with significant human impact. Thus, we chose Cape Shirreff, Livingston Island, an Antarctic Specially Protected Area designated mainly to protect the diversity of marine vertebrate fauna, and selected sampling sites with different types of animals coexisting in a relatively small space, and where human presence and impact are negligible. Using 16S rRNA gene analyses through massive sequencing, we assessed the influence of animal concentrations, via their modification of edaphic characteristics, on soil bacterial diversity and composition. The nutrient composition of soils impacted by Antarctic fur seals and kelp gulls was more similar to that of control soils (i.e. soils without visible presence of plants or animals), which may be due to the more active behaviour of these marine animals compared to other species. Conversely, the soils from concentrations of southern elephant seals and penguins showed greater differences in soil nutrients compared to the control. In agreement with this, the bacterial communities of the soils associated with these animals were most different from those of the control soils, with the soils of penguin colonies also possessing the lowest bacterial diversity. However, all the soils influenced by the presence of marine animals were dominated by bacteria belonging to *Gammaproteobacteria*, particularly those of the genus *Rhodanobacter*. Therefore, we conclude that the modification of soil nutrient composition by marine vertebrates promotes specific groups of bacteria, which could play an important role in the recycling of nutrients in terrestrial Antarctic ecosystems.

## Introduction

Ice-free environments in Antarctica include diverse habitats, ranging from nutrient rich soils in coastal areas, to oligotrophic soils in deserts and high elevation sites. Though most terrestrial environments in Antarctica are subjected to severe environmental conditions, soils harbour abundant and diverse bacterial communities (e.g. [1–6]) and a proportion of bacterial species appear to be unique [7].

Marine vertebrates, such as seabirds and pinnipeds, often colonise ice-free areas on the Antarctic coastline. These animals transfer significant amounts of nutrients and contaminants from the marine ecosystem to the terrestrial ecosystem [8–10], through their faeces, eggs, prey, carcasses, among others [11–14]. Depositions from marine vertebrates strongly influence the physicochemical properties of the soil [15–17], forming terrestrial ecosystems enriched in nutrients [18–20]; indeed, these soils are described as the largest carbon and nitrogen reservoirs in terrestrial Antarctic ecosystems [21]. Enrichment of Antarctic soils by animal depositions promotes an increase in microbial biomass and enzyme activity in general, and soil respiration and nitrogen mineralisation rates in particular [22–27].

Coastal ice-free areas in maritime Antarctica host concentrations of marine vertebrate colonies (seabirds and pinnipeds), which transfer nutrients from the marine to the terrestrial environment. Therefore our hypothesis states that the soil nutrients rather than other edaphic physicochemical parameters of these areas shape the bacterial community composition of the soils underlying the different marine vertebrate concentrations (i.e. the bacterial composition of soil samples from the centre of the colonies). Therefore, the aim of our study was to assess the impact of marine vertebrates, through the modification of edaphic variables, on soil bacterial diversity and community composition in pristine ice-free Antarctic areas.

Several studies have assessed bacterial diversity in Antarctic soils influenced by marine animals using culture independent approaches, such as fingerprinting techniques [28,29], clone libraries [30] and massive sequencing [6,31–33]. Most of these studies have been conducted in areas around the scientific stations on King George Island in the South Shetland Islands, Western Antarctic Peninsula (WAP), probably because they are more readily-accessed by the scientific community. However, these areas on King George Island have been subjected to significant and ongoing disturbances associated with human presence [34], and thus are not necessarily representative areas in the WAP. It is well known that the impacts of local human activities disturb Antarctic environments [35], either by the mere trampling [36,37] or by the transfer of non-indigenous species [38]. Non-indigenous microorganisms could affect Antarctic seabirds and pinnipeds [39,40] and, as they can survive in human waste [41] and mobilise anthropogenic antibiotic resistance [42], different strategies for minimising the spread of infectious diseases have been proposed [43]. Some studies have found that Antarctic soil bacterial communities differ according to the level of human impact in the sampling zone (e.g. [32,44,45]). Moreover, human activities may lead to direct or indirect consequences on animal behaviour and physiology, which could even have a negative impact on their reproduction and survival [35].

An area of the South Shetland Islands with far less human presence and high marine vertebrate diversity is Cape Shirreff, Livingston Island. In Cape Shirreff, some vertebrate species have been subjected to long term scientific monitoring (e.g. [46–50]). The main reason for the designation of this area as an Antarctic Specially Protected Area was to protect the large and diverse seabird and pinniped populations [51]. These features make Cape Shirreff an excellent site for the study of the impact of marine vertebrate concentrations on soil microorganisms, where the presence and impact of human activities are minimal.

## Materials and methods

### Study site and soil sampling

Soil samples were collected during the 48^th^ Antarctic Scientific Expedition (January 2012) with the logistical support and permission of the Chilean Antarctic Institute, at Cape Shirreff (62°27'30"S, 60°47'17"W), Livingston Island, South Shetland Islands, WAP. Cape Shirreff soils are porous and consist mainly of volcanic ash and scoria [52], which support a sparse vegetation, including Antarctic hairgrass (*Deschampsia antarctica*) and mosses as well as some lichens, fungi and macroalgae [51]. This area was designated as 'specially-protected' mainly to safeguard the diversity of animal life, which includes the largest Antarctic fur seal (*Arctocephalus gazella*) breeding colony in the WAP [50], a substantial population of southern elephant seals (*Mirounga leonina*) [53], and a diverse avifauna comprising kelp gulls (*Larus dominicanus*) [54] as well as colonies of gentoo (*Pygoscelis papua*) and chinstrap (*P*. *antarctica*) penguins [55]. The most recent available data on animal population sizes indicate that: (1) Antarctic fur seal colonies reached around 21,000 individuals in the period 2003–2004; (2) the maximum number of southern elephant seals during the 2010–2011 season was 221 individuals; (3) kelp gull nests registered 25 breeding pairs in 2000 along the entire coastline of the area; and (4) a total of 655 gentoo and 3,302 chinstrap penguin nests were registered in 2015–2016, conforming 19 active penguin breeding subcolonies [51].

**Fig 1:**
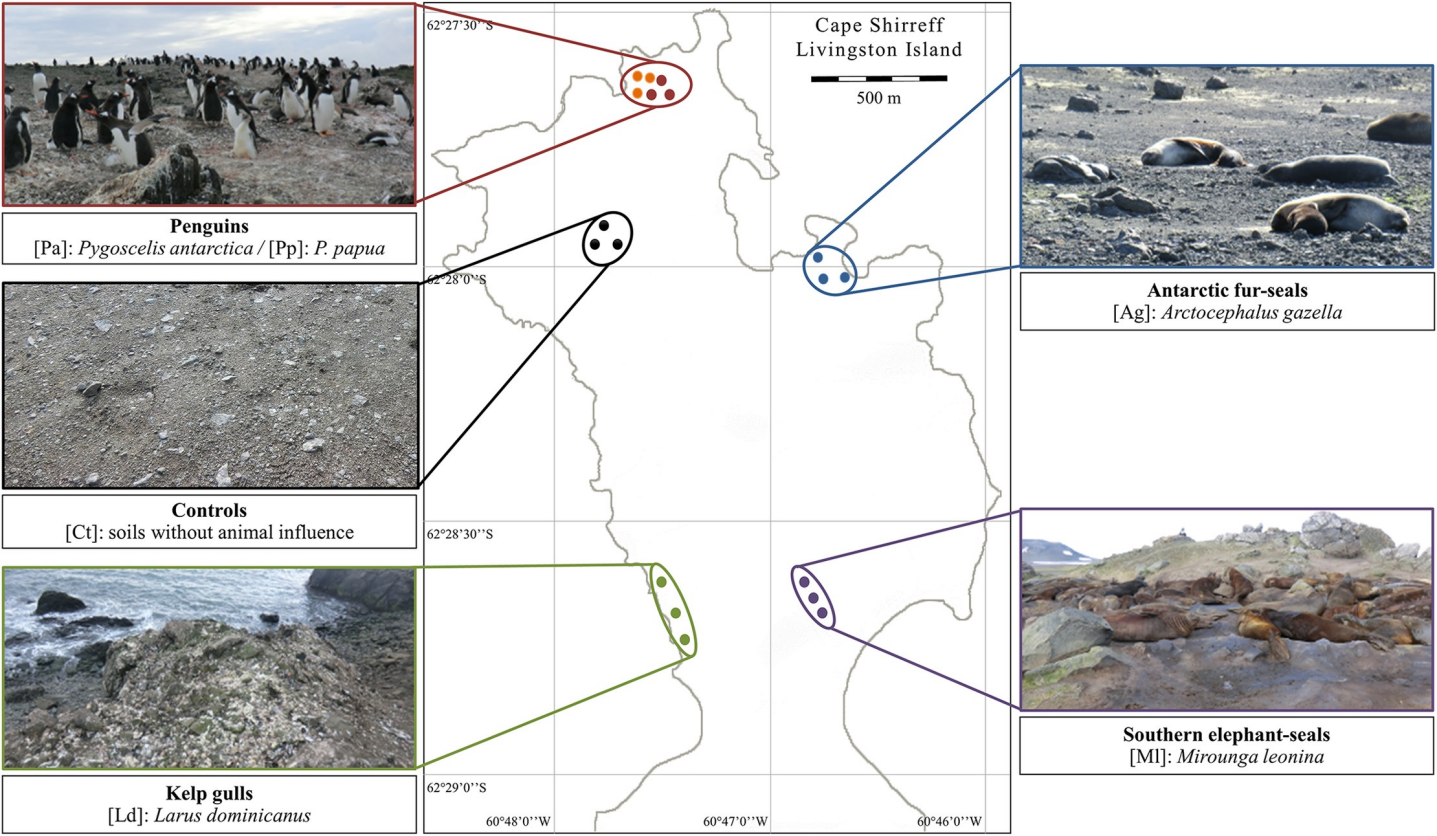
Map of the sampling sites of animal settlement soils at Cape Shirreff (Livingston Island, Antarctica, 62°27'30"S, 60°47'17"W).

In this area, three soil samples of about 50 g each were collected from surface to 10 cm depth from the centre of concentrations of each of the following marine vertebrates: Antarctic fur seals (*A*. *gazella* [Ag]), southern elephant seals (*M*. *leonina* [Ml]), kelp gulls (*L*. *dominicanus* [Ld]) and penguins (*P*. *antarctica* [Pa] and *P*. *papua* [Pp]). In addition, three control [Ct] soil samples of about 50 g each without visible presence of plants or animals were collected in the surrounding area and distant from the vertebrate concentrations to reduce the likelihood of animal influence (Fig 1 and S1 Table). The three samples of each treatment were considered as biological replicates. Sterile disposable implements were used for each sampling and the samples were stored independently in hermetically sealed bags and transported in coolers at 4°C, for about 2 weeks. In the laboratory, the samples were homogenized, sieved through a 2–mm mesh and stored at -20°C until further analyses (about 2 months). A subsample of each treatment was used for DNA extraction and another for the measurement of edaphic variables.

### Extraction of soil DNA and PCR amplification of 16S rRNA gene

DNA was extracted from a 0.25 g subsample of each soil sample using the PowerSoil DNA isolation kit (MoBio) according to the manufacturer’s instructions. The DNA was eluted in 50 μl TE buffer (10 mM Tris-HCl [pH 8.0], 1 mM EDTA) and stored at -20°C until analysis. The concentration and quality of the DNA were determined electrophoretically in 0.8% (w/v) agarose gels in TAE 1X buffer (40 mM Tris-acetate, 1 mM EDTA [pH 8.0]) and visualised by GelRed (Biotium) staining.

The primers used for amplification of the variable regions V1-V3 of the bacterial 16S rRNA gene were A17 and 519R [56]. For pyrosequencing, the "A" sequence (5'-CCATCTCATCCCTGCGTGTCTCCGAC-3') was added via a linker to the forward primer, in addition to a barcode (MID1-MID18) specific for each sample; likewise, the "B" sequence (5'-CCTATCCCCTGTGTGCCTTGGCAGTC-3') was added via a linker to the reverse primer.

All amplifications were performed according to Kumar et al. [56], using the GoTaq Green Master Mix (GoTaq DNA polymerase in 1X Green GoTaq Reaction Buffer [pH 8.5], 200 μM of each dNTP and 1.5 mM MgCl_2_) (Promega) in a Maxygene thermocycler (Axygen). The quality of amplicons was determined by electrophoresis in 1.2% (w/v) agarose gels in 1X TAE buffer stained with GelRed (Biotium). PCR products were then purified using the UltraClean PCR Clean-up kit (MoBio), according to the manufacturer’s instructions. DNA concentration was quantified using an Epoch Micro-Volume Spectrophotometer (Biotek).

### Pyrosequencing of 16S rRNA gene

Equimolar concentrations of purified amplicons were pooled and pyrosequenced using a 454 GS Junior System (Roche) (Centre for Genomics and Bioinformatics, Universidad Mayor, Chile). The sample [Ag]3 could not be included in this analysis because of amplification problems. The reads obtained were analysed using mothur [57]. Briefly, the command *trim*.*seq* was used to classify the sequences by their barcode and to remove the primer and barcode sequences. In addition, sequences with a length of ≤ 200 bp and ≥ 550 bp and an average quality score ≤ 25 in a stepwise (1 bp) moving window of 50 bp were eliminated from the set. Subsequently, sequences were aligned using the command *align*.*seqs* and then denoised using the *pre*.*cluster* command, removing 1,758 sequences from the analysis. Putative chimeric sequences were identified and removed using the command *chimera*.*uchime*.

The remaining good quality sequences were deposited into the NCBI Sequence Read Archive (SRA) database (SRA accession: SRP119005) and aligned with those from the SILVA database v. 115 [58]. Then, a distance matrix was generated by the command *dist*.*seqs* and sequences were clustered in OTUs with 97% sequence identity. The taxonomy of each OTU was assigned using the command *classify*.*otu* and the default command template.

### Measurement of edaphic variables

For each soil sample, the following edaphic factors were measured using standard protocols [59]: pH, water content (WC), organic matter (OM), nitrogen content from ammonium (N-NH_4_^+^) and nitrate (N-NO_3_^–^), and bioavailable phosphorus content (P-Bray).

The pH was determined potentiometrically from 1:10 (w/v) soil:water extracts using a pH electrode connected to a pH 500 meter (Oakton). The water content (WC) was determined from the weight of soil samples before (moist weight, MW) and after (dry weight, DW) drying at 65°C for 24 h according to: WC = ((MW-DW)/DW)*100. The content of organic matter (OM) was determined from the weight of the dry soil samples calcined at 400ºC during 16 h (calcined weight, CW) multiplied by a factor of 0.8 according to: OM = (((DW-CW)/DW)*100)*0.8. The nitrogen content from ammonium (N-NH_4_^+^) was determined from 1:10 (w/v) soil:water extracts using a selective ion electrode connected to an Ion 510 meter (Oakton). The nitrogen content from nitrate (N-NO_3_^-^) was determined by a colorimetric method involving electrophilic aromatic substitution (nitration) between nitronium and salicylate [60] by measuring its absorbance at 410 nm. The phosphorus content (P-Bray) was determined by the extraction of Bray 1 [61] and was detected by the generation of phosphomolybdate blue [62] by measuring its absorbance at 882 nm.

### Statistical and multivariate analyses

For the following analyses we used two datasets: (i) a dataset including all the obtained OTUs (i.e. 1,023 OTUs), and (ii) a dataset including OTUs represented by at least 20 reads when considering all the samples (i.e. the most abundant OTUs). The first dataset was used for taxonomic identification, Venn diagrams and dbRDA analysis, whilst the second dataset was used for cluster-heatmap graphs, the comparison of the abundance of OTUs in samples from vertebrate concentrations vs control samples, and for the correlation-based network analysis. In the case of the clustering, we also include as supplementary material the dendrogram obtained with the first dataset.

First, the richness (S), evenness (J) and Shannon diversity (H') indices were calculated for each sample using the software PAST v3.20 [63]. The pyrosequencing data were Box-Cox transformed prior to the clustering and ordering analyses using the same software.

The clustering of the soil samples according to the edaphic parameters and to the bacterial communities was performed in the software PAST [63], under the unweighted pair-group average (UPGMA) algorithm with Sørensen-Dice distance. Nodes of the dendrograms were tested by bootstrapping with 10,000 replicates. The resultant trees were exported in newick format and imported into the iTOL v4.2.3 platform [64]. In the case of the clustering of the most abundant OTUs, to compare their abundance across the different soil samples, a heatmap analysis was carried out.

Venn diagrams of the OTUs obtained from the different samples were generated according to Heberle et al. [65]. Since diagrams relating 6 types of samples are complicated to visualise, in parallel we analysed the datasets joining the OTUs from both penguin colonies to create the diagram. In addition, correlation-based network analysis was tested by calculating all possible Pearson’s rank correlations between the OTUs. The nodes in the reconstructed networks represent the OTUs, whereas the edges (connections) correspond to a positive or negative strong and significant correlation (Pearson's r>0.7) between the nodes. These analyses were carried out using the packages *vegan* and *qgraph* in R v3.4.4 (http://www.r-project.org).

Permutational multivariate analysis of variance (PERMANOVA) was used to test for significant differences between soil samples from vertebrate concentrations and those of the control soil samples, using Bray Curtis similarity matrices of biological (pyrosequencing) and environmental (edaphic) data. The significance was computed by permutation of group membership, with 10,000 replicates. Pair-wise tests were conducted *post hoc* to determine significant differences among groups following significant PERMANOVA results. All *post hoc* multiple comparison tests were adjusted using a Holm-Bonferroni correction and were performed in the software PAST [63]. Finally, in order to relate the structure of the bacterial communities with the edaphic variables, distance-based redundancy analyses (dbRDA) were performed on Bray Curtis distance using the function *capscale* and the permutation tests using the function *anova* from the package *vegan* in R.

Statistically-significant differences of edaphic variables, diversity indices and OTU abundances between the settlement and control soil samples were determined by non–overlapping 95% confidence intervals of measurements after 10,000 random re–samplings conducted using the *boot* package in R.

## Results

### Bacterial diversity and community composition

Pyrosequencing resulted in 60,530 reads, which after quality and chimera filtering yielded 12,540 sequences. Considering 97% similarity, these bacterial sequences were clustered into 1,023 OTUs. The most abundant phyla in all the soil samples were *Proteobacteria* (50.8% on average), followed by *Actinobacteria* (13.5%), *Bacteroidetes* (10.6%) and *Acidobacteria* (6.8%). In addition, a significant number of reads were identified as unclassified bacteria (8.7%) (Table 1). Of note was that in the control soils, the *Gemmatimonadetes* represented 11.2% of the reads on average, while in the soils of animal settlements, this phylum was less abundant, representing just 0.6% of the reads. Conversely, the *Proteobacteria* phylum was significantly more abundant in the soils of all vertebrate concentrations (except Antarctic fur seals [Ag]) than in the control soils [Ct]. Furthermore, the soils of penguin colonies [Pa and Pp] also had lower abundances of *Actinobacteria*, *Planctomycetes* and unclassified OTUs than the control soils (Table 1).

**Table 1:** Percentage of reads identified at the bacterial phyla level in the soil samples underlying vertebrate concentrations.

		Ct			Ag				Ml				Ld				Pa				Pp			
1	*Acidobacteria*	13.2	±	3.5	8.4	±	4.9		2.3	±	2.1	*	3.7	±	0.8	*	3.9	±	3.7		9.7	±	5.5	
2	*Actinobacteria*	17.5	±	4.3	22.6	±	20.2		21.0	±	3.4		14.9	±	5.0		4.5	±	1.6	*	3.4	±	2.8	*
3	*Armatimonadetes*	0.6	±	0.3	0.0	±	0.0		0.2	±	0.2		0.1	±	0.1		0.0	±	0.0		0.0	±	0.0	
4	*Bacteroidetes*	6.7	±	2.4	3.6	±	1.0		9.0	±	2.6		13.7	±	2.7		18.7	±	7.3		9.6	±	6.0	
5	*Chlorobi*	0.0	±	0.0	0.0	±	0.0		0.0	±	0.0		0.0	±	0.0		0.0	±	0.0		0.1	±	0.1	
6	*Chloroflexi*	0.6	±	0.2	24.4	±	21.3		0.9	±	0.6		0.8	±	0.6		0.0	±	0.0		0.1	±	0.1	
7	*Cyanobacteria/Chloroplasts*	0.6	±	0.3	1.6	±	1.2		0.0	±	0.0		0.2	±	0.1		0.0	±	0.0		0.0	±	0.0	
8	*Deinococcus-Thermus*	0.0	±	0.0	0.4	±	0.4		1.2	±	1.0		0.1	±	0.1		4.9	±	2.6		1.3	±	1.2	
9	*Firmicutes*	1.1	±	0.4	1.2	±	1.0		1.9	±	0.1	*	0.2	±	0.0	*	1.1	±	0.2		0.3	±	0.3	
10	*Gemmatimonadetes*	11.2	±	1.7	0.7	±	0.1	*	0.4	±	0.1	*	1.6	±	0.5	*	0.2	±	0.1	*	0.2	±	0.2	*
11	*Nitrospira*	1.1	±	0.6	0.0	±	0.0		0.0	±	0.0		1.1	±	1.0		0.0	±	0.0		0.0	±	0.0	
12	*Planctomycetes*	0.8	±	0.3	0.9	±	0.6		0.4	±	0.3		1.0	±	0.9		0.1	±	0.1	*	0.1	±	0.1	*
13	*Proteobacteria*	31.5	±	5.7	25.3	±	2.8		53.3	±	4.2	*	50.5	±	5.8	*	62.6	±	8.6	*	73.3	±	8.8	*
14	*TM7_incertae_sedis*	0.0	±	0.0	0.2	±	0.1		0.1	±	0.1		0.0	±	0.0		0.1	±	0.1		0.0	±	0.0	
15	*Verrucomicrobia*	0.0	±	0.0	0.0	±	0.0		0.0	±	0.0		0.1	±	0.1		0.0	±	0.0		0.0	±	0.0	
16	Unclassified	14.9	±	2.4	11.2	±	1.0		9.1	±	3.2		11.9	±	6.0		3.8	±	1.6	*	1.9	±	1.1	*

Values ± standard error are shown. In the same row, values that are statistically–significantly different relative to the controls are indicated by an asterisk (non–overlapping 95% confidence intervals).

Ct: Control, Ag: *Arctocephalus gazella*, Ml: *Mirounga leonina*, Ld: *Larus dominicanus*, Pa: *Pygoscelis antarctica* and Pp: *P*. *papua*.

The most abundant classes were *Gammaproteobacteria* (38.5%) and *Alphaproteobacteria* (6.2%) from *Proteobacteria*, *Sphingobacteria* (4.9%) and *Flavobacteria* (3.9%) from *Bacteroidetes*, and the group Gp1 from *Acidobacteria* (4.1%) (S2 Table).

**Fig 2:**
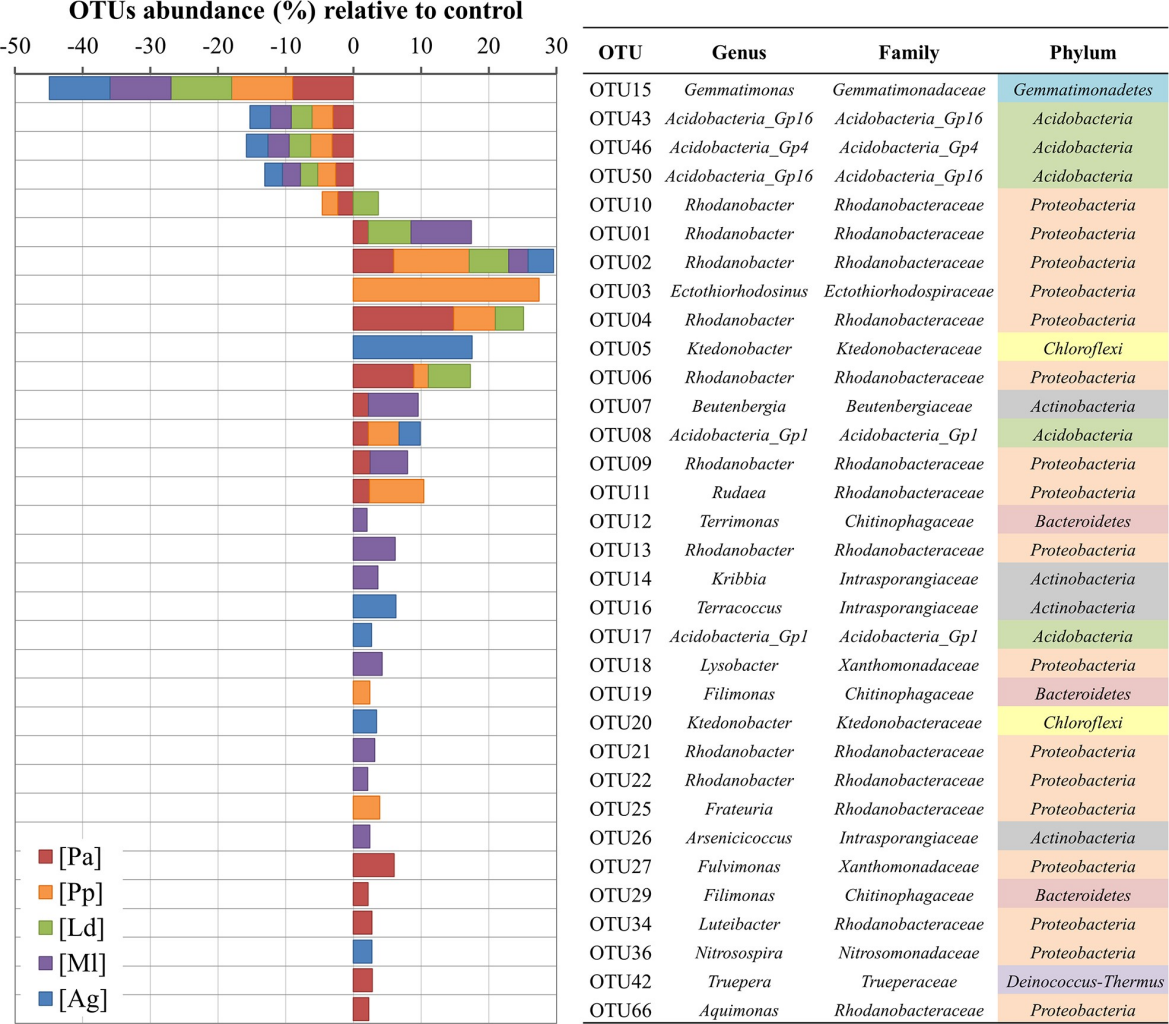
Abundances relative to the control of the OTUs obtained by pyrosequencing of each of the vertebrate concentration soil samples. Only the most abundant OTUs are shown, along with their taxonomic identification. [Ag], *Arctocephalus gazella*; [Ml], *Mirounga leonina*; [Ld], *Larus dominicanus*; [Pa], *Pygoscelis antarctica* and [Pp], *P*. *papua*.

At the genus level, in the control soils, the most abundant OTUs were identified as *Gemmatimonas* from *Gemmatimonadetes* and groups Gp4 and Gp16 from *Acidobacteria* (Fig 2). On the other hand, in most soils from the vertebrate concentrations, the most abundant OTU was identified as *Rhodanobacter* from the *Gammaproteobacteria*, followed by *Ectothiorhodosinus*, in the soils of the penguin colonies (Fig 2).

**Fig 3:**
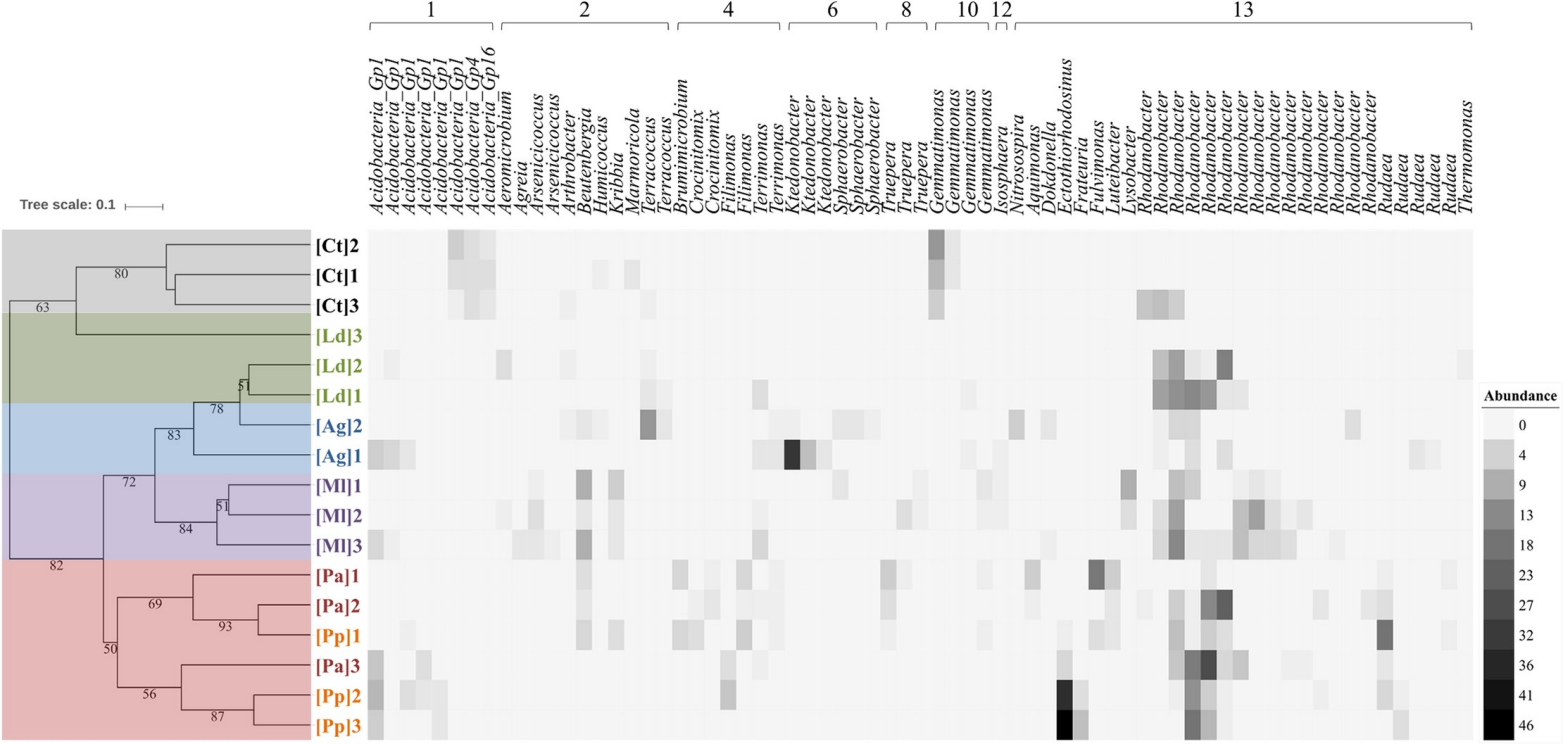
Clustering analysis considering the most abundant OTUs obtained by pyrosequencing and based on the Sørensen-Dice similarity coefficient. Bootstrap support > 50% over 10,000 repeats is shown in the corresponding nodes. Heat map shows the percent relative abundance of the most abundant OTUs. Soil samples: [Ct], Control; [Ag], *Arctocephalus gazella*; [Ml], *Mirounga leonina*; [Ld], *Larus dominicanus*; [Pa], *Pygoscelis antarctica* and [Pp], *P*. *papua*. The number after each abbreviation designates the biological replicate.

The soils of penguin colonies had lower Shannon diversity indices than the control soils, while the latter presented the highest bacterial diversity of all the soils compared (Table 2). Regarding the OTU cluster analyses, both dendrograms, i.e. the clustering obtained with the most abundant OTUs (Fig 3) and the clustering based on the entire sequencing dataset (S1 Fig), show the same groups, probably due to the very low abundance of rare taxa. The soil samples from the penguin [Pa and Pp] colonies and southern elephant seal [Ml] concentrations were grouped into well-defined clusters, with bootstrap support of 50% and 84%, respectively, in the case of the dataset considering the most abundant OTUs. In addition, control samples also formed a definite cluster, with bootstrap support of 80% (Fig 3). The heatmap confirms that the main differences between control soils [Ct] and soils from vertebrate concentrations were due to the more abundant presence of some species of *Acidobacteria* and *Gemmatimonas* in the former and several OTUs related to *Rhodanobacter* in the latter (Fig 3).

**Table 2:** Diversity indices based on pyrosequencing data of bacteria from vertebrate concentration soil samples.

	Ct			Ag			Ml				Ld			Pa				Pp			
**Richness (S)**	136.0	±	27.5	123.0	±	7.3	137.7	±	8.2		153.7	±	45.2	70.0	±	7.4	*	60.0	±	27.5	
**Evenness (J)**	0.893	±	0.010	0.758	±	0.078	0.789	±	0.023	*	0.826	±	0.057	0.754	±	0.064	*	0.667	±	0.056	*
**Shannon (H’)**	4.3	±	0.2	3.7	±	0.4	3.9	±	0.1		4.1	±	0.5	3.2	±	0.3	*	2.6	±	0.5	*

Values ± standard error are shown. In the same row, values that are statistically–significantly different relative to the controls are indicated by an asterisk (non–overlapping 95% confidence intervals).

Ct: Control, Ag: *Arctocephalus gazella*, Ml: *Mirounga leonina*, Ld: *Larus dominicanus*, Pa: *Pygoscelis antarctica* and Pp: *P*. *papua*.

Richness (S) considers the number of OTUs; evenness (J), the distribution of sequences in each OTU; and the Shannon diversity (H') relates the number of different OTUs and how evenly the sequences are distributed among them.

**Fig 4:**
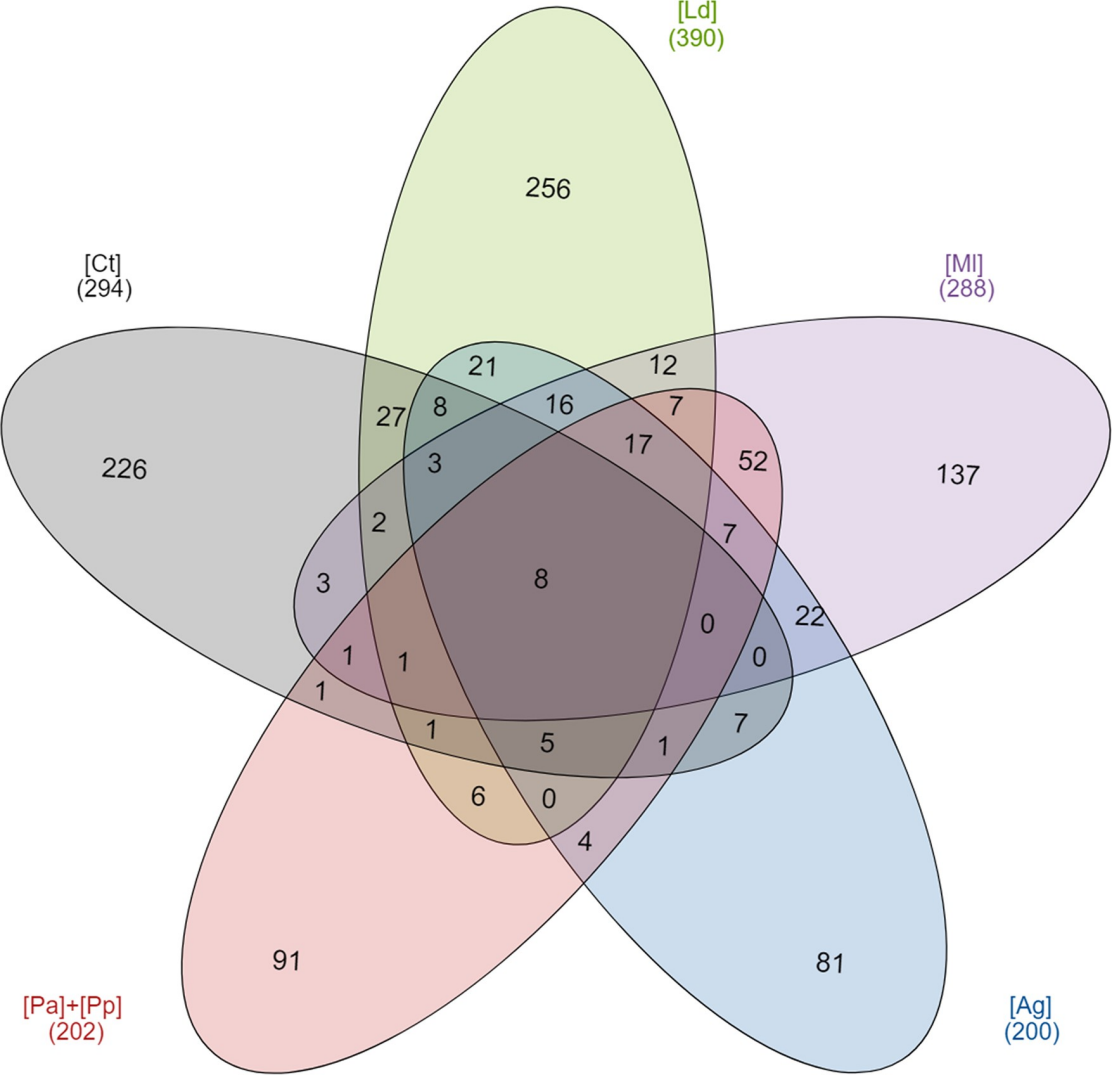
Venn diagram displaying the degree of overlap of bacterial OTUs among the 5 soil types. Soil samples: [Ct], Control; [Ag], *Arctocephalus gazella*; [Ml], *Mirounga leonina*; [Ld], *Larus dominicanus*; [Pa], *Pygoscelis antarctica* and [Pp], *P*. *papua*.

Venn diagrams show that the OTUs obtained differed between the different soil types (S2 Fig illustrates the six soil types whilst Fig 4 includes the data of both penguin species as a single set). Only 7 of the 1,023 OTUs were common to all six soil types, rising to 8 OTUs when the datasets from both penguin colonies were analysed together. Of these, 5 OTUs were related to *Rhodanobacter*, the bacterial genus which was represented by the most abundant OTUs in most soils from the animal settlements.

The number of specific OTUs per soil type ranged from 226 in the control samples [Ct] to 32 or 34 in the penguin [Pa] and [Pp] colonies, respectively. These included over 75% of the OTUs from the control samples and were found exclusively in this soil type. Conversely, besides presenting the lowest number of OTUs (141 for [Pa] and 140 for [Pp]), only ~20% of these OTUs from the penguin colonies were exclusive to those soils. Between them, the penguin colonies shared 25 OTUs, but there were no OTUs common to the soil taken from both penguin colonies and the control soils. Soils from kelp gull [Ld] colonies presented the highest richness of OTUs, with 65% of exclusive OTUs. The remaining OTUs were shared mainly with the soils of the pinnipeds (66 OTUs with [Ml] and 78 OTUs with [Ag]) and the control (55 common OTUs). Finally, the number of OTUs shared between soil types when comparing pinnipeds was higher (73 OTUs) than when comparing seabirds (35 OTUs), and the soils of vertebrate concentrations that shared more OTUs with the control samples [Ct] were Antarctic fur seals [Ag] and kelp gulls [Ld], with 32 and 55 OTUs in common with the control, respectively.

**Fig 5:**
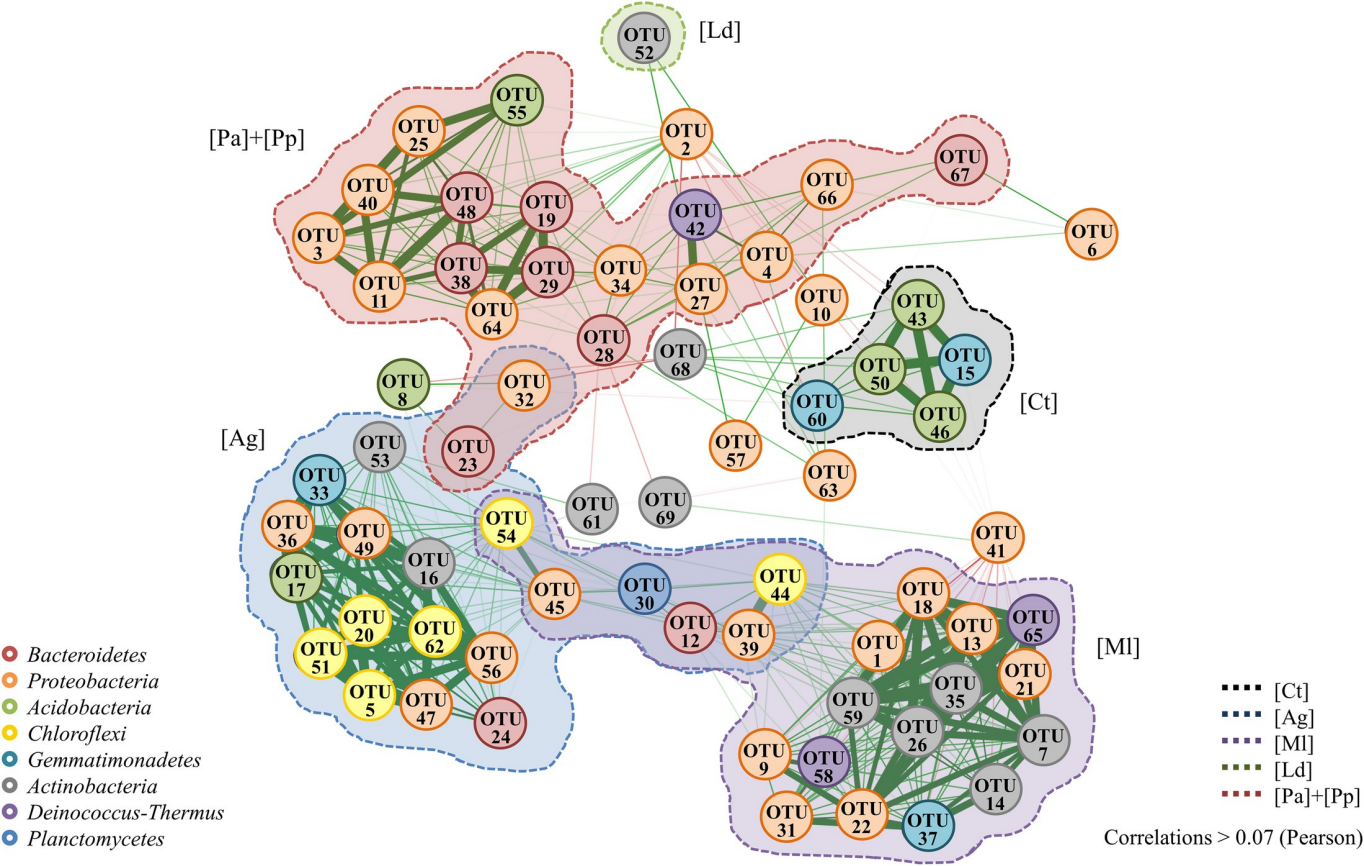
Network analysis based on Pearson correlations considering the most abundant OTUs obtained by pyrosequencing. The circles of the different colors indicate the taxonomic identification of each OTU (see Fig 2). The shaded shapes with dotted edges indicate the soil samples where the OTUs were more abundant: [Ct], Control; [Ag], *Arctocephalus gazella*; [Ml], *Mirounga leonina*; [Ld], *Larus dominicanus*; [Pa], *Pygoscelis antarctica* and [Pp], *P*. *papua*.

Correlation-based network analysis from the pyrosequencing data considering the most abundant OTUs indicated that they were mostly grouped according to the soil type and showed mainly positive correlations (Fig 5).

Specifically, strong positive correlations were observed between the abundance of OTUs identified as *Proteobacteria* (mainly of the *Rhodanobacteraceae* family) and different bacterial groups according to the soil type, i.e. with OTUs related to *Bacteroidetes* in penguin soils, with OTUs related to *Chloroflexi* in the case of soils of Antarctic fur seals [Ag], and with OTUs related to *Actinobacteria* in the soils of Southern elephant seals [Ml]. In the control soils, the OTUs related to *Gemmatimonadetes* and *Acidobacteria* appeared strongly correlated. One exception was the bacterial community in the soils of kelp gulls [Ld], whose OTUs showed weak correlations. In addition, mainly two OTUs (2 and 41), both related to *Rhodanobacter*, showed negative correlations with many other OTUs (Fig 5).

### Soil characterization by edaphic factors

Organic matter and water content were not statistically different between the soils underlying vertebrate concentrations and the control [Ct] soils, while pH was significantly lower only in the soils from Antarctic fur seals [Ag] and one of the penguin [Pp] colonies compared to the control samples. However, ammonium, nitrate and phosphorus contents were higher in the soils of penguin [Pa and Pp] colonies and southern elephant seal [Ml] concentrations (Table 3). Among the vertebrate concentration soils, those of Antarctic fur seals [Ag] had the lowest values of nitrogen from ammonium and nitrate, while the soils of kelp gull [Ld] colonies contained the least bioavailable phosphorus (Table 3). In a cluster analysis of the samples according to these parameters, the soils from the penguin [Pa and Pp] colonies were the most similar to each other (bootstrap = 90%), and were also very similar to the soils of the southern elephant seal [Ml] concentrations (S3 Fig). Conversely, the soils of Antarctic fur seal [Ag] concentrations were similar to the control soil samples (bootstrap = 73%), while those of the kelp gull [Ld] colonies were the least similar to the other vertebrate-influenced soil samples (S3 Fig).

**Table 3:** Edaphic factors of the soil samples collected from vertebrate concentrations.

	Ct				Ag				Ml				Ld				Pa				Pp			
**pH**	6.1	±	0.1		5.4	±	0.2	*	6.5	±	0.4		6.8	±	0.5		6.3	±	0.7		5.2	±	0.2	*
**WC**	17.4	±	2.8		6.9	±	3.9		11.4	±	4.1		14.2	±	3.0		20.5	±	5.0		19.2	±	2.6	
**OM**	9.7	±	6.1		13.3	±	4.2		6.8	±	1.7		9.4	±	3.2		21.1	±	3.4		32.4	±	15.1	
**N-NH**_**4**_^**+**^	6.9	±	3.4		24.8	±	13.4		515.2	±	232.7	*	81.6	±	54.5		752.9	±	200.9	*	371.8	±	61.1	*
**N-NO**_**3**_^**-**^	49.6	±	5.3		59.0	±	37.1		300.2	±	142.6	*	233.4	±	83.7	*	465.3	±	96.6	*	483.7	±	72.2	*
**P-Bray**	1.2	±	0.5		8.0	±	4.4		9.7	±	2.0	*	3.6	±	1.5		43.5	±	3.9	*	39.1	±	1.7	*

Values ± standard error are shown. In the same row, values that are statistically–significantly different relative to the controls are indicated by an asterisk (non–overlapping 95% confidence intervals).

Ct: Control, Ag: *Arctocephalus gazella*, Ml: *Mirounga leonina*, Ld: *Larus dominicanus*, Pa: *Pygoscelis antarctica* and Pp: *P*. *papua*.

WC: water content (%), OM: organic matter (%), N-NH_4_^+^: nitrogen content from ammonium (μg/g) and N-NO_3_^–^: nitrate (μg/g), and P-Bray: bioavailable phosphorus content (μg/g).

### Correlation between bacterial diversity and edaphic factors

PERMANOVA analyses were performed to test if bacterial community composition, edaphic factors and the combination of both (constrained data) were different when comparing the data from the control soil samples with those from the soil samples underlying the different marine vertebrate concentrations (grouping at the levels of “animal species” and “animal types”).

Considering pyrosequencing data, differences in bacterial communities were significant when the factors “animal species” (*p*≤0.1 in all cases) and “animal type” (*p*≤0.01 for seabirds and *p*≤0.05 for pinnipeds) were analysed (Table 4).

**Table 4:** PERMANOVA *p*-values of the data from soil samples underlying vertebrate concentrations when compared to controls.

	**Ag**		**Ml**		**Ld**		**Pa**		**Pp**		**Pinnipeds**		**Seabirds**	
**Pyrosequencing data**	0.0980	**.**	0.0985	**.**	0.0991	**.**	0.0998	**.**	0.0973	**.**	0.0163	*	0.0043	**
**Edaphic data**	0.4003		0.0987	**.**	0.2036		0.0997	**.**	0.0993	**.**	0.1587		0.0094	**
**Constrained data**	0.0988	**.**	0.0991	**.**	0.0995	**.**	0.0988	**.**	0.0982	**.**	0.0486	*	0.0053	**

.*p*≤0.1

* *p*≤0.05

** *p*≤0.01.

Ag: *Arctocephalus gazella*, Ml: *Mirounga leonina*, Ld: *Larus dominicanus*, Pa: *Pygoscelis antarctica* and Pp: *P*. *papua*. Pinnipeds: Ag + Ml; Seabirds: Ld + Pa + Pp.

When comparing the edaphic factors, significant differences were observed only for soils from penguin [Pa and Pp] colonies and southern elephant seal [Ml] concentrations (*p*≤0.1) when grouping by “animal species”, and for soils from seabirds (*p*≤0.01) when grouping by “animal type” (Table 4).

Finally, when bacterial communities analysed by pyrosequencing were constrained by the edaphic factors, the soils from all the vertebrate concentrations (*p*≤0.1) and both seabirds and pinnipeds (*p*≤0.01 and *p*≤0.05, respectively) were significantly different from the control soils (Table 4).

**Fig 6:**
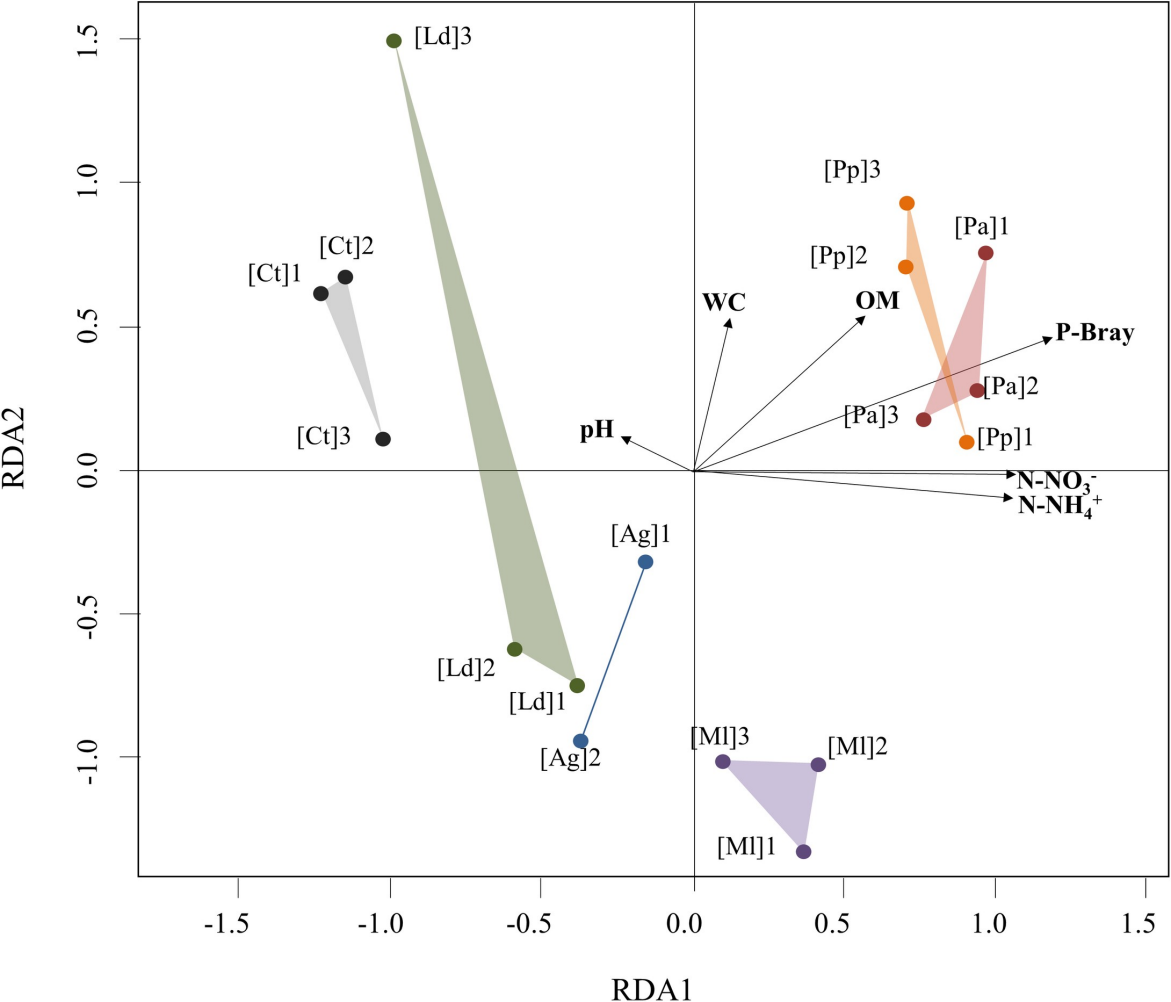
Distance-based redundancy analysis (dbRDA) of pyrosequencing data. Soil samples: [Ct], Control; [Ag], *Arctocephalus gazella*; [Ml], *Mirounga leonina*; [Ld], *Larus dominicanus*; [Pa], *Pygoscelis antarctica* and [Pp], *P*. *papua*. The number after each abbreviation designates the biological replicate. Edaphic variables: pH, water content (WC), organic matter (OM), nitrogen content from ammonium (N-NH_4_^+^) and nitrate (N-NO_3_^–^), and bioavailable phosphorus content (P-Bray).

In order to analyse the effect of the edaphic factors on the structure of the bacterial communities from the different soils, dbRDA analyses were performed using pyrosequencing data (Fig 6). Control soils clearly differed from the soils of vertebrate concentrations (50.4% constrained; 49.6% unconstrained). In the latter, an additional separation was observed, as soils influenced by penguin [Pa and Pp] colonies and southern elephant seal [Ml] concentrations presented the most different bacterial community structure compared to the control soils [Ct] (Fig 6). Conversely, soils influenced by the kelp gull [Ld] and the Antarctic fur seal [Ag] concentrations possessed a bacterial community structure that was intermediate between that of the control soils and the other vertebrate-influenced soils (Fig 6). This structuring of the bacterial communities within the different soil samples was significantly correlated to the pH, the organic matter (OM) and the phosphorus (P-Bray), ammonium (N-NH_4_^+^) and nitrate (N-NO_3_^-^) contents, with the latter accounting most significantly to the variance (*p*≤0.01) (Table 5).

**Table 5:** Redundancy coefficients and *p*-values of correlations between edaphic factors and both axes of dbRDA analysis of pyrosequencing data.

	RDA1	RDA2	*p*-value	
**pH**	-0.1906	0.0882	0.087	**.**
**WC**	0.0945	0.4185	0.186	
**OM**	0.4435	0.4260	0.096	**.**
**N-NH**_**4**_^**+**^	0.8055	-0.0714	0.067	**.**
**N-NO**_**3**_^**-**^	0.8146	-0.0073	0.002	**
**P-Bray**	0.9207	0.3600	0.090	**.**
**Total Variance**	39.4%	21.2%	0.002	**

.*p*≤0.1

** *p*≤0.01.

WC: water content (%), OM: organic matter (%), N-NH_4_^+^: nitrogen content from ammonium (μg/g) and N-NO_3_^–^: nitrate (μg/g), and P-Bray: bioavailable phosphorus content (μg/g).

## Discussion

Ice-free zones in Antarctica are subjected to low temperatures, and their soils typically exhibit low levels of moisture, carbon and nitrogen, as well as low buffering capacity [30]. However, close to Antarctic coastlines, animal deposition is a key determinant of the soil nutrient composition, enriching it in nitrogen and total organic carbon [28], among other key elements for sustaining soil bacterial communities. Our results support the hypothesis that different soil bacterial compositions are associated with different marine vertebrate concentrations due to the habitat properties related to soil nutrient composition.

To the best of our knowledge, there are no previous studies describing bacterial communities in Antarctic soils underlying vertebrate concentrations from Cape Shirreff on Livingston Island (South Shetland Islands), a site where human activity has been strictly restricted to scientific research. Conversely, in Fildes Peninsula at King George Island, also located in the South Shetland Islands, several studies have described the influence of concentrations of penguins and other birds [31,32,48] as well as pinnipeds [66] on these Antarctic soils; however in most of these analyses, human impact cannot be discarded.

Different species of marine vertebrates have their own gastrointestinal microbiota [67–69] and are vectors that transfer biogenic compounds from the sea onto land in Antarctica, thus influencing soil properties [10,15,16,18–21,70]. A previous study in soils of ice-free areas of Livingston Island reported a strong influence of nutrients on the bacterial community structure, reporting that moss-covered soils favoured *Bacteroidetes*, while oligotrophic soils were dominated by *Acidobacteria* [3]. Accordingly, we observed that soils enriched by animal wastes also sustain high abundance of *Bacteroidetes*, *Actinobacteria* and *Chloroflexi*, although they are dominated by *Gammaproteobacteria*, a bacterial group common to all soils from vertebrate concentrations. In contrast, *Acidobacteria* was more abundant in control soils than in soils underlying these concentrations. Representatives of the phylum *Acidobacteria* have been reported to be better adapted to low nutrient conditions [71]. On the other hand, we found that the phylum *Gemmatimonadetes*, in particular the genus *Gemmatimonas*, was less represented in the soils associated with marine vertebrates. This genus is highly abundant in Antarctic soils with low amounts of nutrients and water scarcity [1].

The genus *Rhodanobacter* (*Gammaproteobacteria*) was the most abundant in soils from vertebrate concentrations. Eighteen species have currently been described as belonging to this genus (www.bacterio.net/rhodanobacter.html), most of them isolated from diverse soil environments. This genus includes slow-growing bacteria with an anaerobic facultative metabolism; in the absence of oxygen, they grow using nitrate, nitrite and nitrous oxide as electron acceptors and exhibit complete denitrification [72,73]. This bacterial genus, and others from the *Rhodanobacteraceae* family, were previously identified in other Antarctic soils colonized by penguins [28,30,31], suggesting that they could be adapted to environments with a high concentration of nutrients. Additionally, *Rhodanobacter* was also identified in non-Antarctic soils with high nitrate contents and slightly low pH [74]. Some *Rhodanobacter* use amino acids rather than sugars for growth and may grow under nitrate-reducing conditions [75]. In our study, we found that OTUs related to *Rhodanobacteraceae* exhibited strong correlations with OTUs related to *Bacteroidetes* (in [Pa] and [Pp] soils), *Actinobacteria* (in [MI] soils) and *Chloroflexi* (in [Ag] soils); therefore, members of these families could establish important relationships in soils enriched in nitrate such as those associated with marine vertebrates.

Interestingly, in the correlation-based network, a single cluster (i.e. densely interconnected nodes) was associated with each soil type, which could represent a unique microbial community whose OTUs could be related to functional associations within the community. Moreover, there were no strong correlations between the clusters of each type of soil suggesting that bacteria were able to establish associations with specific partners according to the environmental conditions of each soil. Correlation networks of co-occurring microorganisms are usually used for the prediction of species interactions in environments ranging from soils to oceans [76–78]. In this sense, when two species (or any taxonomically relevant unit) co-occur or show a similar abundance pattern over multiple samples (positive correlation), a positive relationship is usually assumed; conversely, when they show mutual exclusion (negative correlation), a negative relationship is predicted [79]. However, interpretation of these ecological relationships is far from easy [80]. On the one hand, mutualism, commensalism and competence are normally well detected by most networking tools, but parasitism, amensalism and syntrophy are normally undetectable or incorrectly detected. On the other hand, deductions derived from applying network analyses to microbial relationships can vary depending on the taxonomic level chosen and the criteria used to build the networks. Therefore, as interpretation of such networks as indicators of ecological relationships is not unequivocal, we prefer just to describe co-occurrence when clusters (i.e. densely interconnected nodes) connecting OTUs from the same type of soil were observed. In addition to predictions of individual relationships among microorganisms, the association structure of the correlation-based network may provide insights into the organisation of microbial communities [79]. For example, organisms with a high degree of interaction with others may be key in their community, and thus their disturbance or elimination could greatly affect the community structure [77]. The co-occurrence patterns observed between OTUs related to *Rhodanobacter* and OTUs of different bacterial families depending on the type of soil, in addition to the denitrification potential of members of this genus, suggest that *Rhodanobacter* species could play an important role in these microbial communities enriched in animal wastes, and could be considered keystone species of these communities [81].

The bacterial diversity in the soils of Cape Shirreff, as indicated by the diversity indices, was comparable with that obtained by Kim et al. [31], who reported low Shannon diversity indices in penguin ornithogenic soils. Wang et al. [32] found higher diversity indices, although they did not find significant differences in the Shannon diversity indices between the soils of penguin colonies and pristine soils (without animal influence).

In our multivariate analyses, control soils differed from soils of the vertebrate concentrations, both when grouped at the “animal type” level and at the “animal species” level. The soils influenced by penguins and southern elephant seals presented the most different bacterial community structure compared to the control soils. This could be because these animals are more sedentary or site-specific, and so occupy locations more intensely compared to the kelp gulls and Antarctic fur seals [82,83]. However, the effects that each vertebrate concentration has on the biological and physicochemical composition of the soil depend on several factors, such as differences in diet, excretion, behaviour, among others.

Birds mainly excrete uric acid as a waste product, in contrast with mammals that excrete primarily urea [84]; however, the degradation of both compounds enriches the soils in ammonium which could then be transformed into nitrate by nitrification. In addition, animal diet and foraging behaviour could also contribute to the observed differences since they affect the chemical composition of bird guano and mammalian faeces. The diet of penguins and Antarctic fur seals is dominated by Antarctic krill (*Euphausia superba*) [85,86], but various fish species have also been occasionally recorded in their diet [86,87]. In the case of southern elephant seals, considered as top predators in Antarctic marine ecosystems, the analysis of stomach samples showed that cephalopods were the main prey, followed by fish [88], which could explain the distinctness of their associated soils from the rest of the soil samples. Finally, an analysis of regurgitated pellets indicated that kelp gulls on the South Shetland Islands consumed predominantly intertidal prey, principally *Nacella concinna* [89]. However kelp gulls are a predatory, commensal and opportunistic species, consistent with their associated soil samples presenting the greatest variability.

In general, we observed the highest impact on soil biological and physicochemical composition in the case of penguin colonies, which are associated with large guano deposits [90]. This is in accordance with the data of Wang et al. [32], which suggested that seals may impose less impact on the soil bacterial community than penguins. In our study we included soil samples from colonies of two penguin species, and although they show differences in phenology [91] and foraging strategies [92], we did not observe significant differences in the impact that their colonies had on the soil.

In order to determine if the differences of the bacterial communities between soils underlying vertebrate concentrations and the control soils were dependent on habitat properties, we measured different edaphic factors. The pH was significantly lower in the soils associated with Antarctic fur seals and one of the penguin colonies compared to the control soils. In previous studies, pH has been measured in different layers of ornithogenic (i.e. bird-formed) soils; the superficial layer is neutral whilst deeper layers are slightly acidic [66]. The samples of the present work correspond to superficial soil, therefore the values obtained for this parameter agree with those of previous studies [31,32,66]. In addition, we found that higher amounts of inorganic nutrients like nitrate, ammonium and phosphate were generally found in the Antarctic soils influenced by marine vertebrates, compared to control soils, which is in general agreement with previous reports from Fildes Peninsula soils [28,31,32]. This was mainly observed in soils influenced by penguin colonies, where we found significantly more inorganic nutrients than in the control soils without vertebrate influence. The high amount of nutrients has a strong effect on the cryptogamic flora, impairing the growth of vegetation in the close vicinity [66], and as shown here, also diminishing the diversity of soil bacterial communities. Penguins feed on krill, which contain a lot of fluorine that is concentrated in their guano, and is a very important phosphate-forming element in Antarctic ornithogenic soils [93] along with other sources of phosphorus, like phosphine [94]. As phosphorus in Antarctic ornithogenic soils inhibits microbial growth [24], this could explain the low diversity found in the soils of penguin colonies, which thus only support the growth and activity of specialised groups of bacteria.

Soils formed by the influence of vertebrate concentrations are typically abundant in depositions such as bird guano and mammalian faeces, and are generally rich in nitrogen and total carbon [28]. These physicochemical parameters are those that mainly influence the structure of the dominant bacterial communities in soils of Livingston Island [3], and not pH as in temperate soils [95]. Indeed, although a full comparison between our results and those of Ganzert et al. [3] is difficult, because the latter study did not include vertebrate concentrations as drivers of soil bacterial communities, we found that almost all measured edaphic factors were significant in structuring the soil microbial communities, with the nitrogen content from nitrate having the greatest influence.

Although bacterial growth is inhibited in Antarctic soils enriched in vertebrate depositions, the activity of certain enzymes related to nutrient cycling seems to be favoured [24,27], which may affect emissions of greenhouse gases such as CH_4_ and N_2_O in this region [18,19,96]. This is of particular concern if we consider reports that the populations of some marine animals have been increasing [97,98], since if these changes affect greenhouse gas emissions, they could reverse the apparent temporary regional cooling trend in the WAP since the late-1990s [99]. Therefore, it is important to increase knowledge of microbial diversity in such soils, especially in pristine areas where soil microbial diversity has not been assessed previously, such as in Cape Shirreff on Livingston Island, an Antarctic Specially Protected Area mainly designated to protect marine vertebrate diversity.

## Supporting information

S1 Fig Clustering analysis considering the OTUs obtained by pyrosequencing and based on the Sørensen-Dice similarity coefficient. Bootstrap support over 50% of 10,000 repeats is shown in the corresponding nodes. Soil samples: [Ct], Control; [Ag], *Arctocephalus gazella*; [Ml], *Mirounga leonina*; [Ld], *Larus dominicanus*; [Pa], *Pygoscelis antarctica* and [Pp], *P*. *papua*. The number after each abbreviation designates the biological replicate.
(TIF)

S2 Fig Venn diagram displaying the degree of overlap of bacterial OTUs among the 6 soil types. Soil samples: [Ct], Control; [Ag], *Arctocephalus gazella*; [Ml], *Mirounga leonina*; [Ld], *Larus dominicanus*; [Pa], *Pygoscelis antarctica* and [Pp], *P*. *papua*.
(TIF)

S3 Fig Clustering analysis considering the edaphic parameters and based on the Sørensen-Dice similarity coefficient. Soil samples: [Ct], Control; [Ag], *Arctocephalus gazella*; [Ml], *Mirounga leonina*; [Ld], *Larus dominicanus*; [Pa], *Pygoscelis antarctica* and [Pp], *P. papua*.
(TIF)

S1 Table Geo-referencing data of the soil samples underlying animal settlements. (DOCX)

S2 Table Percentage of reads identified at bacterial subphyla level in the soil samples underlying animal settlements. (DOCX)
