# Supplementary figures and images for: Seabird and pinniped shape soil bacterial communities of their settlements in Cape Shirreff, Antarctica
Source: PLoS One. 2019 Jan 9;14(1):e0209887. doi: 10.1371/journal.pone.0209887 (PMC6326729; doi:10.1371/journal.pone.0209887)

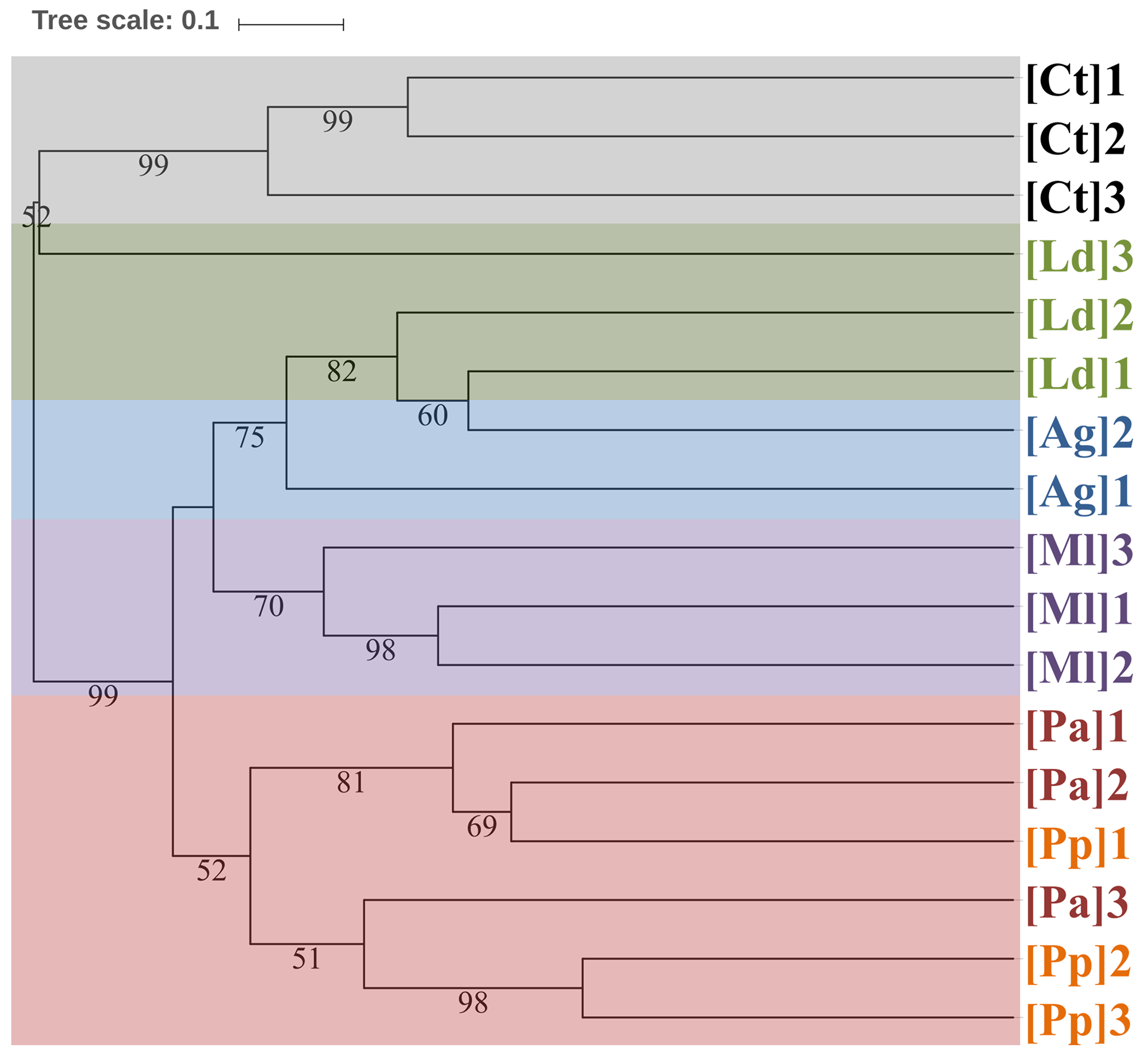

Supplement: S1 Fig — Bootstrap support over 50% of 10,000 repeats is shown in the corresponding nodes. Soil samples: [Ct], Control; [Ag], Arctocephalus gazella; [Ml], Mirounga leonina; [Ld], Larus dominicanus; [Pa], Pygoscelis antarctica and [Pp], P. papua. The number after each abbreviation designates the biological replicate. (TIF) [file pone.0209887.s001.tif]

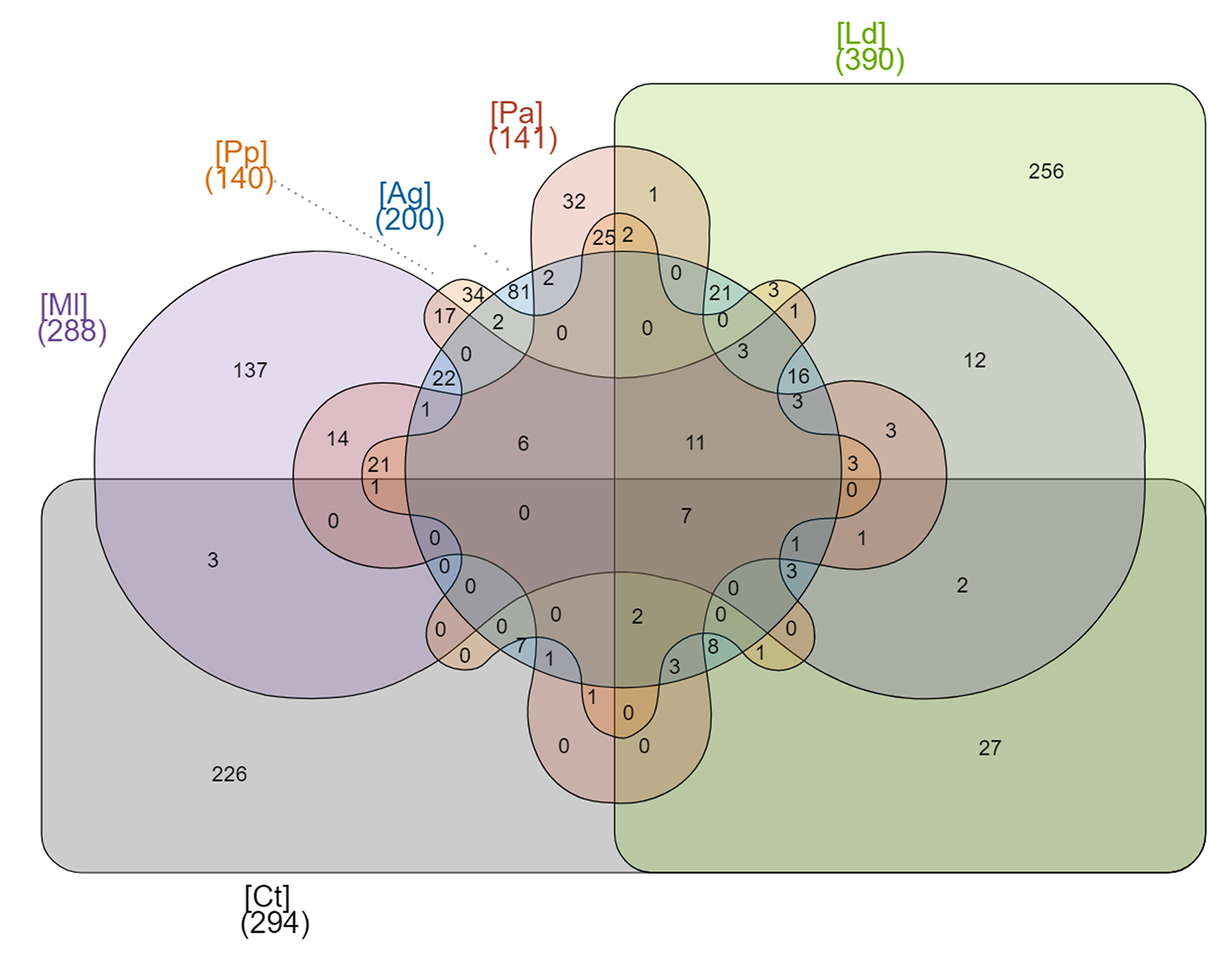

Supplement: S2 Fig — Soil samples: [Ct], Control; [Ag], Arctocephalus gazella; [Ml], Mirounga leonina; [Ld], Larus dominicanus; [Pa], Pygoscelis antarctica and [Pp], P. papua. (TIF) [file pone.0209887.s002.tif]

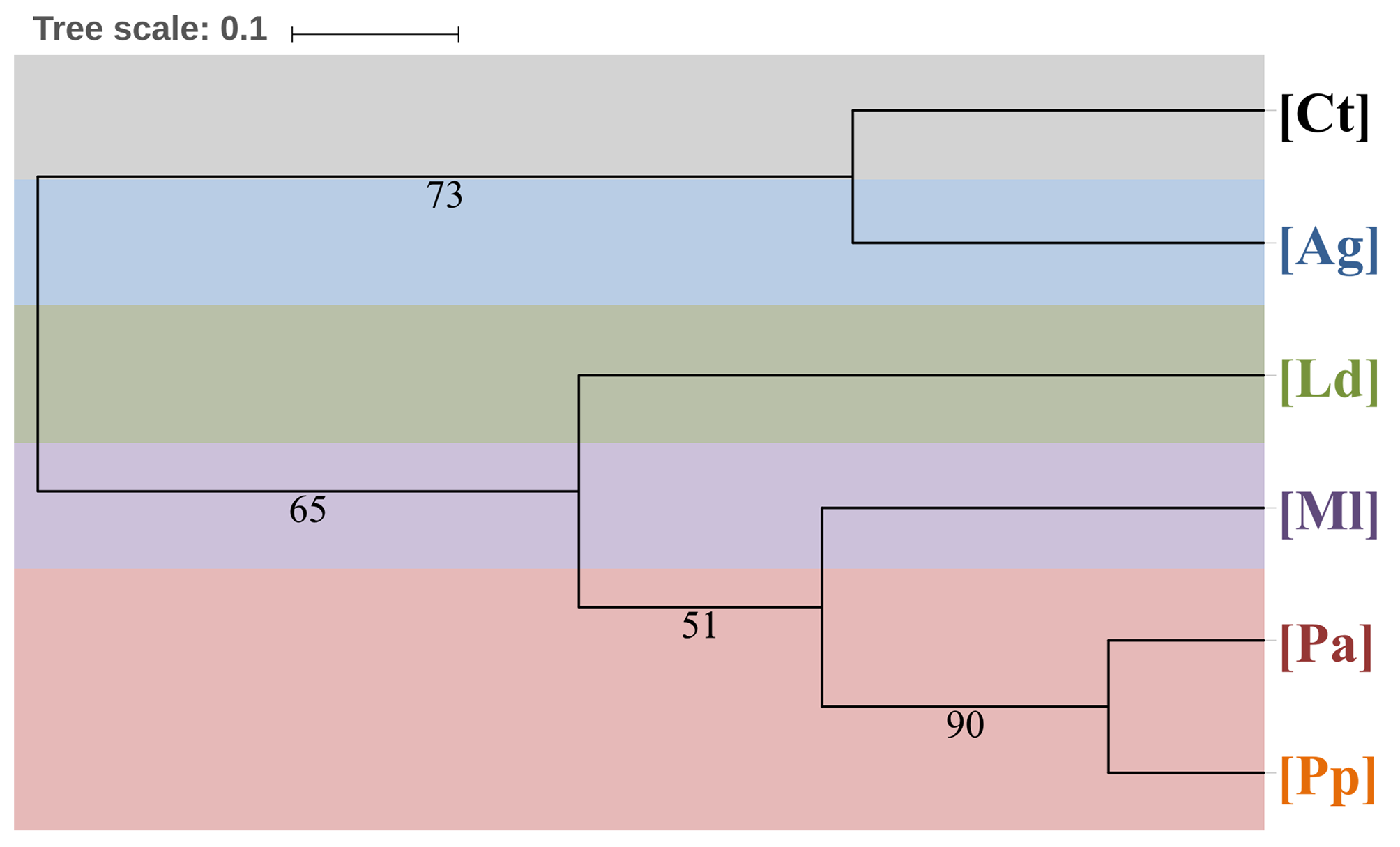

Supplement: S3 Fig — Clustering analysis considering the edaphic parameters and based on the Sørensen-Dice similarity coefficient. Soil samples: [Ct], Control; [Ag], Arctocephalus gazella; [Ml], Mirounga leonina; [Ld], Larus dominicanus; [Pa], Pygoscelis antarctica and [Pp], P. papua. (TIF) [file pone.0209887.s003.tif]
