# Supplementary material for: Seabird and pinniped shape soil bacterial communities of their settlements in Cape Shirreff, Antarctica
Source: PLoS One. 2019 Jan 9;14(1):e0209887. doi: 10.1371/journal.pone.0209887 (PMC6326729; doi:10.1371/journal.pone.0209887)
Supplement: S1 Table — (DOCX) [file pone.0209887.s004.docx]

**S1 Table.** Geo-referencing data of the soil samples underlying animal settlements.

|  | Latitude | Longitude | Altitude |
| --- | --- | --- | --- |
| Ct1 | S62°27.910´ | W60°47.631´ | 22 m |
| Ct2 | S62°27.901´ | W60°47.637´ | 25 m |
| Ct3 | S62°27.894´ | W60°47.605´ | 23 m |
| Ag1 | S62°28.016´ | W60°46.464´ | 59 m |
| Ag2 | S62°28.017´ | W60°46.665´ | 22 m |
| Ag3 | S62°28.052´ | W60°46.698´ | 37 m |
| Ml1 | S62°28.678´ | W60°46.756´ | 15 m |
| Ml2 | S62°28.649´ | W60°46.802´ | 12 m |
| Ml3 | S62°28.608´ | W60°46.861´ | 9 m |
| Ld1 | S62°28.790´ | W60°47.373´ | 7 m |
| Ld2 | S62°28.774´ | W60°47.416´ | 6 m |
| Ld3 | S62°28.589´ | W60°47.482´ | 4 m |
| Pa1 | S62°27.656´ | W60°47.286´ | 16 m |
| Pa2 | S62°27.646´ | W60°47.302´ | 16 m |
| Pa3 | S62°27.645´ | W60°47.378´ | 10 m |
| Pp1 | S62°27.626´ | W60°47.363´ | 6 m |
| Pp2 | S62°27.624´ | W60°47.391´ | 8 m |
| Pp3 | S62°27.657´ | W60°47.398´ | 16 m |

Ct: Control, Ag: *Arctocephalus gazella*, Ml: *Mirounga leonina*, Ld: *Larus dominicanus*, Pa: *Pygoscelis antarctica* and Pp: *P. papua*.
