# Supplementary material for: Seabird and pinniped shape soil bacterial communities of their settlements in Cape Shirreff, Antarctica
Source: PLoS One. 2019 Jan 9;14(1):e0209887. doi: 10.1371/journal.pone.0209887 (PMC6326729; doi:10.1371/journal.pone.0209887)
Supplement: S2 Table — (DOCX) [file pone.0209887.s005.docx]

**S2 Table.** Percentage of reads identified at bacterial subphyla level in the soil samples underlying animal settlements.

| **Phyla^a^** | **Subphyla** | **Ct** |  |  | **Ag** |  |  |  | **Ml** |  |  |  | **Ld** |  |  |  | **Pa** |  |  |  | **Pp** |  |  |  |
| --- | --- | --- | --- | --- | --- | --- | --- | --- | --- | --- | --- | --- | --- | --- | --- | --- | --- | --- | --- | --- | --- | --- | --- | --- |
| 1 | *Acidobacteria_Gp1* | 0.4 | ± | 0.2 | 8.1 | ± | 5.2 | * | 2.3 | ± | 2.1 |  | 1.5 | ± | 0.7 |  | 3.9 | ± | 3.7 |  | 9.7 | ± | 5.5 | * |
|  | *Acidobacteria_Gp3* | 0.1 | ± | 0.1 | 0.0 | ± | 0.0 |  | 0.0 | ± | 0.0 |  | 0.1 | ± | 0.1 |  | 0.0 | ± | 0.0 |  | 0.0 | ± | 0.0 |  |
|  | *Acidobacteria_Gp4* | 5.0 | ± | 1.5 | 0.2 | ± | 0.2 | * | 0.0 | ± | 0.0 | * | 1.2 | ± | 0.9 | * | 0.0 | ± | 0.0 | * | 0.0 | ± | 0.0 | * |
|  | *Acidobacteria_Gp5* | 0.1 | ± | 0.1 | 0.0 | ± | 0.0 |  | 0.0 | ± | 0.0 |  | 0.0 | ± | 0.0 |  | 0.0 | ± | 0.0 |  | 0.0 | ± | 0.0 |  |
|  | *Acidobacteria_Gp6* | 1.9 | ± | 0.8 | 0.0 | ± | 0.0 | * | 0.0 | ± | 0.0 | * | 0.3 | ± | 0.3 | * | 0.0 | ± | 0.0 | * | 0.0 | ± | 0.0 | * |
|  | *Acidobacteria_Gp7* | 0.5 | ± | 0.1 | 0.0 | ± | 0.0 | * | 0.0 | ± | 0.0 | * | 0.0 | ± | 0.0 | * | 0.0 | ± | 0.0 | * | 0.0 | ± | 0.0 | * |
|  | *Acidobacteria_Gp10* | 0.0 | ± | 0.0 | 0.0 | ± | 0.0 |  | 0.0 | ± | 0.0 |  | 0.0 | ± | 0.0 |  | 0.0 | ± | 0.0 |  | 0.0 | ± | 0.0 |  |
|  | *Acidobacteria_Gp11* | 0.1 | ± | 0.1 | 0.0 | ± | 0.0 |  | 0.0 | ± | 0.0 |  | 0.0 | ± | 0.0 |  | 0.0 | ± | 0.0 |  | 0.0 | ± | 0.0 |  |
|  | *Acidobacteria_Gp14* | 0.0 | ± | 0.0 | 0.1 | ± | 0.1 |  | 0.0 | ± | 0.0 |  | 0.0 | ± | 0.0 |  | 0.0 | ± | 0.0 |  | 0.0 | ± | 0.0 |  |
|  | *Acidobacteria_Gp16* | 5.1 | ± | 1.0 | 0.1 | ± | 0.1 | * | 0.0 | ± | 0.0 | * | 0.7 | ± | 0.3 | * | 0.0 | ± | 0.0 | * | 0.0 | ± | 0.0 | * |
|  | *Acidobacteria_Gp17* | 0.0 | ± | 0.0 | 0.0 | ± | 0.0 |  | 0.0 | ± | 0.0 |  | 0.0 | ± | 0.0 |  | 0.0 | ± | 0.0 |  | 0.0 | ± | 0.0 |  |
|  | *Acidobacteria_Gp22* | 0.1 | ± | 0.1 | 0.0 | ± | 0.0 |  | 0.0 | ± | 0.0 |  | 0.0 | ± | 0.0 |  | 0.0 | ± | 0.0 |  | 0.0 | ± | 0.0 |  |
| 2 | *Actinobacteria* | 17.5 | ± | 4.3 | 22.6 | ± | 20.2 |  | 21.0 | ± | 3.4 |  | 14.9 | ± | 5.0 |  | 4.5 | ± | 1.6 | * | 3.4 | ± | 2.8 | * |
| 3 | *Armatimonadetes_Gp4* | 0.2 | ± | 0.2 | 0.0 | ± | 0.0 |  | 0.0 | ± | 0.0 |  | 0.0 | ± | 0.0 |  | 0.0 | ± | 0.0 |  | 0.0 | ± | 0.0 |  |
|  | *Armatimonadia* | 0.0 | ± | 0.0 | 0.0 | ± | 0.0 |  | 0.2 | ± | 0.2 |  | 0.1 | ± | 0.1 |  | 0.0 | ± | 0.0 |  | 0.0 | ± | 0.0 |  |
|  | *Chthonomonadetes* | 0.5 | ± | 0.3 | 0.0 | ± | 0.0 |  | 0.0 | ± | 0.0 | * | 0.0 | ± | 0.0 | * | 0.0 | ± | 0.0 | * | 0.0 | ± | 0.0 | * |
| 4 | *Bacteroidetes_incertae_sedis* | 0.1 | ± | 0.1 | 0.0 | ± | 0.0 |  | 0.0 | ± | 0.0 |  | 0.3 | ± | 0.2 |  | 0.0 | ± | 0.0 |  | 0.0 | ± | 0.0 |  |
|  | *Bacteroidia* | 0.1 | ± | 0.1 | 0.0 | ± | 0.0 |  | 0.2 | ± | 0.1 |  | 0.0 | ± | 0.0 |  | 0.0 | ± | 0.0 |  | 0.0 | ± | 0.0 |  |
|  | *Flavobacteria* | 0.5 | ± | 0.1 | 0.0 | ± | 0.0 |  | 4.5 | ± | 3.1 |  | 7.9 | ± | 3.3 |  | 6.5 | ± | 4.0 |  | 2.7 | ± | 2.4 |  |
|  | *Sphingobacteria* | 4.6 | ± | 1.6 | 3.4 | ± | 1.2 |  | 3.4 | ± | 0.9 |  | 4.3 | ± | 0.5 |  | 8.4 | ± | 1.6 |  | 4.9 | ± | 2.5 |  |
|  | Other | 1.5 | ± | 0.7 | 0.2 | ± | 0.2 |  | 1.0 | ± | 0.4 |  | 1.3 | ± | 0.8 |  | 3.9 | ± | 1.8 |  | 1.9 | ± | 1.6 |  |
| 5 | *Ignavibacteria* | 0.0 | ± | 0.0 | 0.0 | ± | 0.0 |  | 0.0 | ± | 0.0 |  | 0.0 | ± | 0.0 |  | 0.0 | ± | 0.0 |  | 0.1 | ± | 0.1 |  |
| 6 | *Anaerolineae* | 0.0 | ± | 0.0 | 0.0 | ± | 0.0 |  | 0.0 | ± | 0.0 |  | 0.4 | ± | 0.4 |  | 0.0 | ± | 0.0 |  | 0.0 | ± | 0.0 |  |
|  | *Caldilineae* | 0.4 | ± | 0.1 | 0.0 | ± | 0.0 | * | 0.0 | ± | 0.0 | * | 0.1 | ± | 0.1 | * | 0.0 | ± | 0.0 | * | 0.0 | ± | 0.0 | * |
|  | *Chloroflexi* | 0.0 | ± | 0.0 | 0.0 | ± | 0.0 |  | 0.0 | ± | 0.0 |  | 0.1 | ± | 0.1 |  | 0.0 | ± | 0.0 |  | 0.0 | ± | 0.0 |  |
|  | *Ktedonobacteria* | 0.2 | ± | 0.2 | 23.6 | ± | 22.0 |  | 0.0 | ± | 0.0 |  | 0.0 | ± | 0.0 |  | 0.0 | ± | 0.0 |  | 0.1 | ± | 0.1 |  |
|  | *Thermomicrobia* | 0.0 | ± | 0.0 | 0.8 | ± | 0.8 |  | 0.9 | ± | 0.6 |  | 0.0 | ± | 0.0 |  | 0.0 | ± | 0.0 |  | 0.0 | ± | 0.0 |  |
|  | Other | 0.0 | ± | 0.0 | 0.0 | ± | 0.0 |  | 0.0 | ± | 0.0 |  | 0.3 | ± | 0.2 |  | 0.0 | ± | 0.0 |  | 0.0 | ± | 0.0 |  |
| 7 | *Chloroplast* | 0.6 | ± | 0.3 | 0.6 | ± | 0.2 |  | 0.0 | ± | 0.0 |  | 0.1 | ± | 0.1 |  | 0.0 | ± | 0.0 |  | 0.0 | ± | 0.0 |  |
|  | *Cyanobacteria* | 0.0 | ± | 0.0 | 1.0 | ± | 1.0 |  | 0.0 | ± | 0.0 |  | 0.1 | ± | 0.1 |  | 0.0 | ± | 0.0 |  | 0.0 | ± | 0.0 |  |
| 8 | *Deinococci* | 0.0 | ± | 0.0 | 0.4 | ± | 0.4 |  | 1.2 | ± | 1.0 |  | 0.1 | ± | 0.1 |  | 4.9 | ± | 2.6 | * | 1.3 | ± | 1.2 |  |
| 9 | *Bacilli* | 0.5 | ± | 0.3 | 0.5 | ± | 0.5 |  | 0.7 | ± | 0.2 |  | 0.1 | ± | 0.1 |  | 0.2 | ± | 0.2 |  | 0.0 | ± | 0.0 |  |
|  | *Clostridia* | 0.6 | ± | 0.6 | 0.8 | ± | 0.6 |  | 1.2 | ± | 0.2 |  | 0.1 | ± | 0.1 |  | 0.9 | ± | 0.4 |  | 0.3 | ± | 0.3 |  |
|  | Other | 0.0 | ± | 0.0 | 0.0 | ± | 0.0 |  | 0.0 | ± | 0.0 |  | 0.0 | ± | 0.0 |  | 0.0 | ± | 0.0 |  | 0.0 | ± | 0.0 |  |
| 10 | *Gemmatimonadetes* | 11.2 | ± | 1.7 | 0.7 | ± | 0.1 | * | 0.4 | ± | 0.1 | * | 1.6 | ± | 0.5 | * | 0.2 | ± | 0.1 | * | 0.2 | ± | 0.2 | * |
| 11 | *Nitrospira* | 1.1 | ± | 0.6 | 0.0 | ± | 0.0 |  | 0.0 | ± | 0.0 |  | 1.1 | ± | 1.0 |  | 0.0 | ± | 0.0 |  | 0.0 | ± | 0.0 |  |
| 12 | *Phycisphaerae* | 0.0 | ± | 0.0 | 0.0 | ± | 0.0 |  | 0.0 | ± | 0.0 |  | 0.3 | ± | 0.3 |  | 0.0 | ± | 0.0 |  | 0.1 | ± | 0.1 |  |
|  | *Planctomycetacia* | 0.8 | ± | 0.3 | 0.9 | ± | 0.6 |  | 0.4 | ± | 0.3 |  | 0.6 | ± | 0.5 |  | 0.1 | ± | 0.1 | * | 0.0 | ± | 0.0 | * |
| 13 | *Alphaproteobacteria* | 16.8 | ± | 1.0 | 4.3 | ± | 0.0 | * | 2.3 | ± | 0.1 | * | 10.3 | ± | 5.5 |  | 1.3 | ± | 0.6 | * | 1.5 | ± | 0.7 | * |
|  | *Betaproteobacteria* | 4.1 | ± | 0.6 | 4.1 | ± | 2.8 |  | 3.1 | ± | 1.3 |  | 3.0 | ± | 0.6 |  | 2.0 | ± | 0.8 |  | 1.4 | ± | 1.2 |  |
|  | *Deltaproteobacteria* | 1.0 | ± | 0.4 | 0.2 | ± | 0.1 | * | 0.2 | ± | 0.1 | * | 1.7 | ± | 1.4 |  | 0.5 | ± | 0.2 |  | 0.2 | ± | 0.2 |  |
|  | *Gammaproteobacteria* | 8.7 | ± | 5.7 | 16.4 | ± | 0.2 |  | 46.4 | ± | 5.5 | * | 34.2 | ± | 12.2 |  | 55.8 | ± | 10.0 | * | 62.3 | ± | 12.0 | * |
|  | Other | 1.0 | ± | 0.2 | 0.5 | ± | 0.1 | * | 1.5 | ± | 0.8 |  | 1.3 | ± | 1.0 |  | 3.0 | ± | 0.6 | * | 7.8 | ± | 1.4 | * |
| 14 | *TM7_incertae_sedis* | 0.0 | ± | 0.0 | 0.2 | ± | 0.1 |  | 0.1 | ± | 0.1 |  | 0.0 | ± | 0.0 |  | 0.1 | ± | 0.1 |  | 0.0 | ± | 0.0 |  |
| 15 | *Opitutae* | 0.0 | ± | 0.0 | 0.0 | ± | 0.0 |  | 0.0 | ± | 0.0 |  | 0.1 | ± | 0.1 |  | 0.0 | ± | 0.0 |  | 0.0 | ± | 0.0 |  |
| 16 | Unclassified | 14.9 | ± | 2.4 | 11.2 | ± | 1.0 |  | 9.1 | ± | 3.2 |  | 11.9 | ± | 6.0 |  | 3.8 | ± | 1.6 | * | 1.9 | ± | 1.1 | * |

^a^ See the phyla corresponding to each number in Table 1.

Values ± standard error are shown. In the same row, values statistically–significantly different regarding the Control are followed by an asterisk (non–overlapping 95% confidence intervals).

Ct: Control, Ag: *Arctocephalus gazella*, Ml: *Mirounga leonina*, Ld: *Larus dominicanus*, Pa: *Pygoscelis antarctica* and Pp: *P. papua*.
